# Curvilinear association between cardiometabolic index and depressive symptoms in individuals aged 45 and older: a cross-sectional study of CHARLS

**DOI:** 10.3389/fpubh.2025.1534302

**Published:** 2025-03-19

**Authors:** Sibo Han, Yingqi Zhang, Bingxin Wu, Qingyun Chen, Zhengyuan Han, Jingmin Chen, Peishan Li, Meimei Xu

**Affiliations:** ^1^Second Clinical Medical College, Guangzhou University of Chinese Medicine, Guangzhou, China; ^2^The Fourth Clinical Medical College of Guangzhou University of Chinese Medicine, Shenzhen, China; ^3^The Second Affiliated Hospital of Guangzhou University of Chinese Medicine, Guangdong Provincial Hospital of Chinese Medicine, Guangzhou, Guangdong, China

**Keywords:** cardiometabolic index, CESD-10, depression, middle-aged adults, older adults, CHARLS

## Abstract

**Objective:**

This research is aimed at investigating the association between the cardiometabolic index (CMI) and depressive symptoms in Chinese population of middle and older age, using data derived from the CHARLS study.

**Methods:**

Using data from 7,800 participants in the 2011–2012 wave of the CHARLS cohort, this cross-sectional analysis concentrated on examining the association between CMI and depressive symptoms, assessed through CESD-10 scores. The study utilized multivariate logistic regression, multiple linear regression, and restricted cubic spline (RCS) models to investigate the link between CMI and depression, with subgroup analyses offering further insights. Sensitivity analyses included propensity score matching and data from 8,457 participants in the 2015–2016 CHARLS wave.

**Results:**

In fully adjusted models, higher CMI was significantly associated with an elevated risk of depression, with participants having a CMI ≥ 0.594 showing a 162% higher risk compared to those with lower CMI. The RCS analysis identified a threshold at CMI = 0.594, where participants with CMI ≥ 0.594 had a 162% elevated possibility of depression in comparison to those with CMI < 0.594 [OR = 2.62, 95% CI: 2.36–2.91]. Sensitivity analyses, including propensity score matching and data from the 2015–2016 CHARLS wave, confirmed the robustness of the findings.

**Conclusion:**

Our analysis demonstrates that elevated CMI levels are independently correlated with a heightened likelihood of experiencing depressive symptoms, highlighting the significance of metabolic interventions in mitigating depressive tendencies in middle-aged and older individuals.

## Introduction

Depression is a widespread psychological disorder that significantly affects personal wellbeing, social dynamics, and economic productivity across the globe (1). Following the COVID-19 epidemic, there has been a sharp rise in the worldwide occurrence of depression, disproportionately affecting middle-aged and older populations (2). A systematic review revealed that 35.1% of older adults worldwide suffer from depression, highlighting an urgent need for targeted interventions (3).

Current treatment options for depression encompass psychotherapy, pharmacotherapy, electrotherapy, and combination therapies, yet many of these approaches have limitations, mainly for the older population. Antidepressant medications are frequently prescribed and show clinical efficacy; however, they are often linked to adverse effects, including increased risks of falls, fractures, and other complications, which may limit their long-term use, particularly in older adults (4).

The pathogenesis of depression is multifactorial, shaped by a complex interplay of genetic, psychological, and environmental factors, as well as biological dysregulations. Genetic factors, including abnormalities in receptor systems, play a critical role in the onset of depression (5). Additionally, psychological stressors and immune system dysfunction, particularly inflammation, have been shown to significantly contribute to the development and progression of depressive symptoms (6). Recent studies have increasingly examined the connection between metabolic health and mental wellbeing, proposing that both may share common pathophysiological pathways (7–9).

Meta-analyses consistently demonstrate that individuals with impaired metabolic health are at a significantly heightened risk of developing depressive symptoms (10). Studies have shown that individuals experiencing depressive symptoms often exhibit distinct alterations in their lipid profiles. Specifically, depression has been associated with lower levels of high-density lipoprotein (HDL) cholesterol and elevated levels of very-low-density lipoprotein cholesterol, triglycerides, and total cholesterol. These lipid profile changes may be indicative of underlying metabolic dysregulation in depression, potentially contributing to the increased risk of cardiometabolic diseases in depressed individuals (10, 11). These findings indicate a robust link between lipid metabolism dysregulation and depressive symptoms, highlighting the potential for metabolic markers as indicators of mental health risk.

Consequently, markers that accurately represent fluctuations in lipid levels could be valuable biomarkers for diagnosing depressive disorders.

The cardiometabolic index (CMI) is figured outperforming the multiplication of the triglyceride to high-density lipoprotein cholesterol (TG/HDL-C) ratio with waist-to-height ratio [WHtR; (12)], indicates both the level of obesity and the individual's lipid metabolic state. As a novel indicator, CMI provides a more comprehensive assessment of cardiometabolic health compared to traditional measures like BMI, as demonstrated in various studies (9, 13, 14), and has already been demonstrated to be useful in identifying diabetes (12) and erectile dysfunction (15), as well as and predicting the progression of atherosclerosis (16) and renal function (17). Although the relationship between CMI and depression has been investigated among US populations (8, 9), evidence from other demographic groups, particularly in non-Western contexts, remains scarce. Our study applies data from the China Health and Retirement Longitudinal Study (CHARLS) to investigate the relationship between CMI and depressive symptoms among adult Chinese individuals who are in their middle age or older.

## Methods and materials

### Study design and population

**Figure 1:**
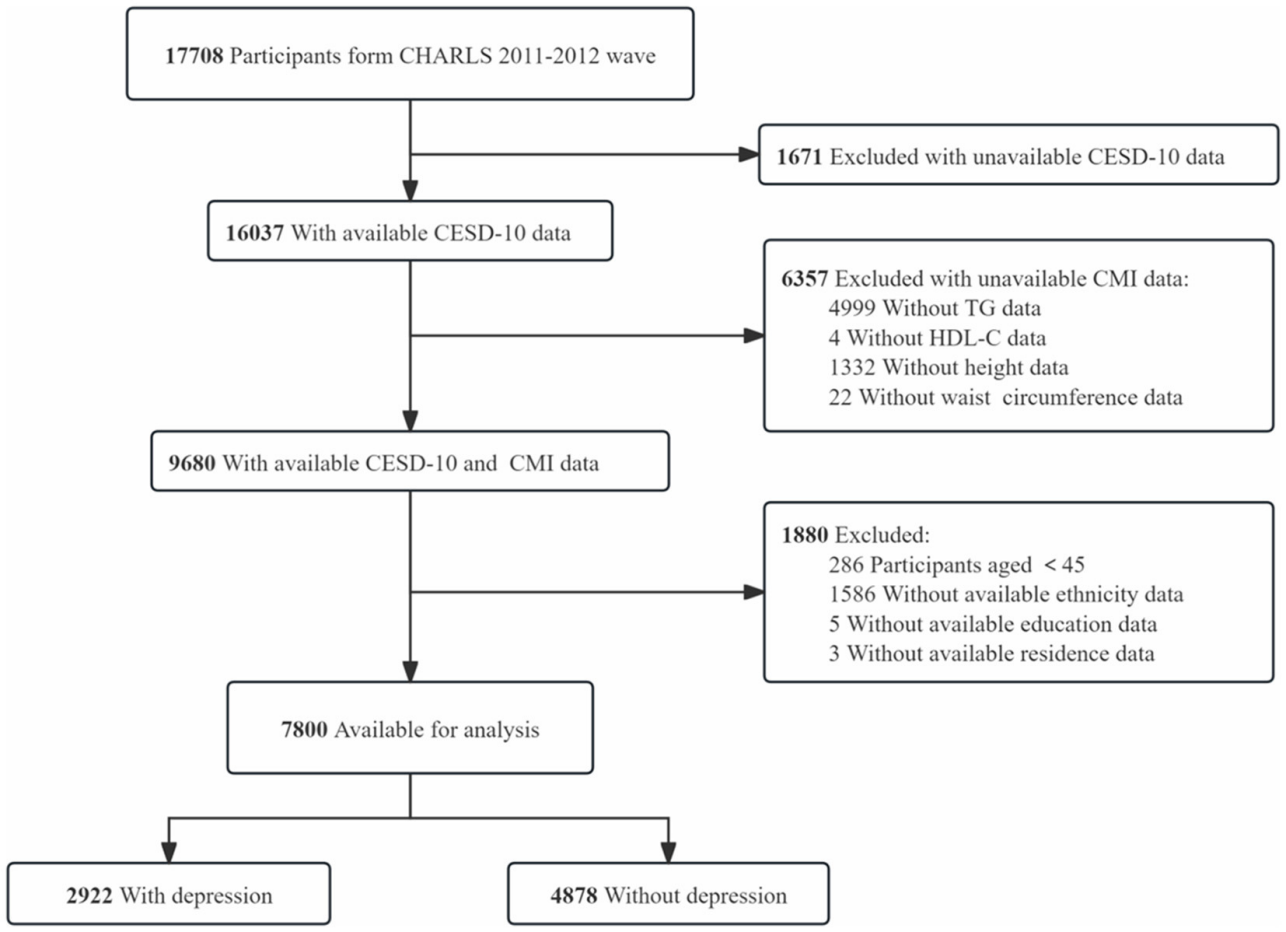
Flow chart of participant selection. This diagram outlines the step-by-step process of participant inclusion and exclusion, from the initial CHARLS dataset to the final sample of 7,800 participants included in the analysis.

This research employed data from the CHARLS (18), a nationwide survey representing Chinese individuals who are 45 years old and older, conducted by the National Institute of Development at Peking University. The CHARLS study was granted approval by the Biomedical Ethics Review Committee at Peking University, and all participants provided written informed consent (IRB00001052-11015). Participants who were younger than 45 years old and those with incomplete essential data, such as CESD-10 responses, height, waist circumference, triglycerides (TG), HDL-C, and essential covariates (e.g., marital status, ethnicity, education, and residence), were excluded. In total, 7,800 participants from the 2011–2012 wave of the CHARLS cohort were incorporated in the final analysis (Figure 1).

### Cardiometabolic index

CMI is calculated using four key variables: triglycerides (TG), high-density lipoprotein cholesterol (HDL-C), height, and waist circumference. The formula is: CMI = TG (mmol/L)/HDL-C (mmol/L) × waist circumference (cm)/height (cm) (8, 9, 12).

In the CHARLS cohort study, blood samples were gathered from participants who signed informed consent forms, and blood samples were collected by experienced medical personnel from participants who provided informed consent (19, 20). Blood samples were collected at designated healthcare facilities and transported to certified laboratories for standardized analysis. Anthropometric measurements were generally recorded by trained interviewers during home visits (20).

Comprehensive details on the study design, data collection procedures, and implementation protocols can be sought on the official CHARLS website (https://charls.pku.edu.cn/wd/a2011nqgjxdc.htm).

### Assessment of depression

Symptoms of depression were evaluated by the 10-item version of the Center for Epidemiological Studies Depression Scale [CESD-10; (21)], a highly validated tool for assessing depression in older populations (22–24). The CESD-10 is made up of 10 questions, each question offers four possible answers to choose from, varying from “rarely or never” to “most or all of the time,” with scores ranging from 0 to 3. The overall CESD-10 score spans from 0 to 30, where superior values signify more severe depressive symptoms. A score of 10 or higher on the CESD-10 was considered indicative of significant depressive symptoms, suggesting clinically relevant depression (21, 25).

### Covariates

Covariates included demographic and health-related factors such as gender (26), age, Han ethnicity, education level (27), residence, marital status, drinking status, smoking status, social activities, hypertension (19), diabetes (28), total cholesterol, low-density lipoprotein, cystatin C, C-reactive protein, creatinine, blood urea nitrogen, and uric acid.

Smoking status was categorized as “never,” “before,” and “current” categories according to participants' feedback to the questions: “Have you ever smoked?” and “Do you currently smoke?” (26). Similarly, drinking status was similarly divided into “never,” “before,” or “current,” according to participants' responses to the questions: “Did you drink alcohol in the past year?” and “Do you currently drink?” (29). Participants were deemed socially active if they participated in more than one social activity in the past 30 days, based on their self-reported participation in social activities. Hypertension was classified as self-reported diagnosis or diastolic blood pressure ≥90 mmHg and/or systolic blood pressure ≥140 mmHg, gauged during household surveys (19). Diabetes was classified as fasting blood glucose ≥7.0 mmol/L, HbA1c ≥6.5%, use of insulin for glycemic control, or self-reported diagnosis (28).

### Statistical analysis

The continuous variables were reported as medians with interquartile ranges (IQR) or means with standard deviations (SD), while the categorical variables were portrayed as frequencies and percentages. The chi-square test aimed to compare categorical data, while the Mann-Whitney U test or Student's *t*-test was utilized for continuous data, depending on suitability. To address missing data, multiple imputation was applied, maintaining the robustness of the results. To investigate the relationship between CMI and depressive symptoms, four logistic regression models were applied with adjustments for relevant covariates, and four linear regression models were developed to further assess the connection between CMI and depression symptoms, adjusting for possible confounding factors.

Subgroup analyses presented that the association between higher CMI and a heightened risk of depression remained consistent across groups categorized by gender, residence type, alcohol consumption, smoking habits, diabetes, and hypertension. To account for baseline differences between covariates, sensitivity analyses using propensity score matching to reckon with baseline differences in covariates. To minimize the potential effect of different survey rounds and further assess the robustness of our results, participants from the 2015–2016 CHARLS wave were included in the sensitivity analyses. Restricted cubic splines (RCS) were employed to investigate possible non-linear relationships between CMI and depression risk. CMI was split into quartiles, and trend *p*-values were calculated to examine CMI as a continuous variable and assess potential non-linear trends. Statistical analyses were conducted utilizing the R (version 4.3.1, R Project for Statistical Computing), with a two-sided *p*-value of < 0.05 regarded as statistically significant (30).

## Results

### Characteristics of the participants

Among the 17,708 participants in the 2011–2012 wave of the CHARLS cohort, 16,037 had CESD-10 questionnaire data, and 9,680 provided fasting blood samples. A total of 286 participants younger than 45 years old, 1,586 participants with missing ethnicity information, 5 participants with missing education data, and 3 participants lacking residence information were excluded, leaving 7,800 participants to be analyzed. Of these, 2,922 participants were classified as having depressive symptoms, while 4,878 participants were classified as not exhibiting depressive symptoms.

The mean age of the 7,800 participants was 58.7 ± 8.7 years (Table 1), with 55.0% being female (*n* = 4,289). According to CESD-10 scores, 2,922 participants (37.5%) were classified as presenting with depressive symptoms. Participants with depressive symptoms had an older average age (59.4 ± 8.7 years) and a higher likelihood of being female (64.8%), live with a partner (85.2%), were more likely to have lower educational attainment (57.3%) and to reside in rural areas (72.2%).

**Table 1:** Baseline characteristics of the study population (*n* = 7,800).

**Variables**	**Total (*n* = 7,800)**	**Non-depressed (*n* = 4,878)**	**Depressed (*n* = 2,922)**	***P***
Gender				< 0.001
Female	4,289 (55.0)	2,396 (49.1)	1,893 (64.8)	
Male	3,511 (45.0)	2,482 (50.9)	1,029 (35.2)	
Age	58.7 ± 8.7	58.2 ± 8.7	59.4 ± 8.7	< 0.001
Marriage				< 0.001
Unmarried	848 (10.9)	415 (8.5)	433 (14.8)	
Married	6,952 (89.1)	4,463 (91.5)	2,489 (85.2)	
Han ethnicity				0.2
No	524 (6.7)	314 (6.4)	210 (7.2)	
Yes	7,276 (93.3)	4,564 (93.6)	2,712 (92.8)	
Education				< 0.001
Below primary school level	3,717 (47.7)	2,044 (41.9)	1,673 (57.3)	
Primary school	1,726 (22.1)	1,101 (22.6)	625 (21.4)	
Junior high school	1,580 (20.3)	1,121 (23)	459 (15.7)	
High school and above	777 (10.0)	612 (12.5)	165 (5.6)	
Residence				< 0.001
Urban community	2,613 (33.5)	1,802 (36.9)	811 (27.8)	
Rural village	5,187 (66.5)	3,076 (63.1)	2,111 (72.2)	
Social activities				< 0.001
Passive	3,832 (49.1)	2,271 (46.6)	1,561 (53.4)	
Positive	3,968 (50.9)	2,607 (53.4)	1,361 (46.6)	
Alcohol consumption				< 0.001
Never	4,630 (59.4)	2,787 (57.1)	1,843 (63.1)	
Before	627 (8.0)	337 (6.9)	290 (9.9)	
Current	2,543 (32.6)	1,754 (36)	789 (27)	
Smoking status				< 0.001
Never	4,845 (62.1)	2,887 (59.2)	1,958 (67)	
Before	626 (8.0)	410 (8.4)	216 (7.4)	
Current	2,329 (29.9)	1,581 (32.4)	748 (25.6)	
Hypertension				0.101
No	4,744 (60.8)	3,001 (61.5)	1,743 (59.7)	
Yes	3,056 (39.2)	1,877 (38.5)	1,179 (40.3)	
Diabetes				0.283
No	6,242 (80.0)	3,922 (80.4)	2,320 (79.4)	
Yes	1,558 (20.0)	956 (19.6)	602 (20.6)	
TC (mg/dl)	194.1 ± 38.9	193.4 ± 38.9	195.3 ± 38.9	0.035
LDL (mg/dl)	116.8 ± 35.1	116.2 ± 35.1	117.7 ± 35.1	0.082
Weight (kg)	59.1 ± 11.6	60.5 ± 11.6	56.9 ± 11.4	< 0.001
BUN (mg/dl)	15.7 ± 4.4	15.7 ± 4.4	15.7 ± 4.4	0.823
Creatinine (mg/dl)	0.8 ± 0.2	0.8 ± 0.2	0.8 ± 0.2	< 0.001
Uric acid (mg/dl)	4.4 ± 1.2	4.5 ± 1.2	4.3 ± 1.2	< 0.001
Cystatin C (mg/dl)	1.0 ± 0.2	1.0 ± 0.2	1.0 ± 0.2	0.077
CRP (mg/dl)	1.0 (0.5, 2.1)	1.0 (0.5, 2.1)	1.0 (0.5, 2.1)	0.739

OR, odds ratio; CI, confidence interval; TC, total cholesterol; LDL, low-density lipoprotein; BUN, blood urea nitrogen; CRP, C-reactive protein.

### Multivariable regression analyses

**Figure 2:**
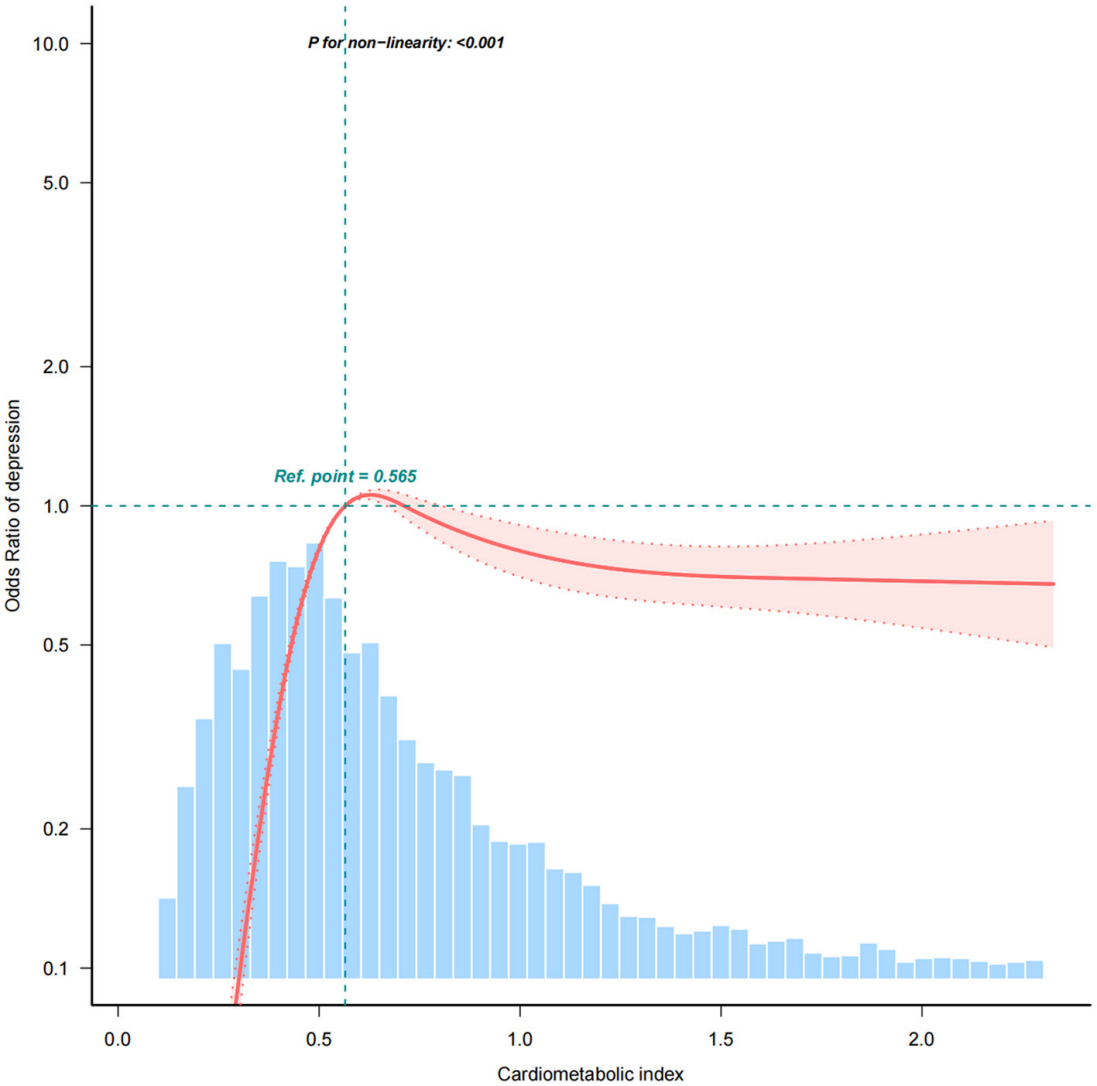
The association between cardiometabolic index (CMI) and depressive symptoms using restricted cubic spline (RCS) regression analysis. The RCS curve demonstrates a non-linear relationship between CMI and the risk of depressive symptoms. A significant threshold was identified at CMI = 0.594 (Supplementary Table 1), above which the risk of depression sharply increases, indicating a 162% elevated depression risk for individuals with CMI ≥ 0.594 compared to those with lower CMI levels. The curve shows how depression risk stabilizes beyond this point.

Table 2 presents the association between CMI and depression. In the unadjusted Model 1, a prominent positive correlation was identified between CMI and CESD-10 scores (β = 0.16, 95% CI: 0.07–0.26). Each unit rise in CMI corresponded to an 8% higher probability of exhibiting depressive symptoms (OR = 1.08, 95% CI: 1.04–1.11). In the fully adjusted Model 4, this association strengthened (β = 0.34, 95% CI: 0.22–0.46), with each unit increase in CMI linked to an 18% higher probability of acquiring depressive symptoms (OR = 1.18, 95% CI: 1.12–1.25). The associations consistently retained statistical significance (*p* for trend < 0.001) after CMI quartiles were converted into categorical variables. In the unadjusted Model 1, participants in Q3 exhibited a 904% increased risk of depression in comparison to those in the lowest quartile (Q1) [OR = 10.04, 95% CI: 8.41–11.99], while those in Q4 demonstrated a 674% higher risk [OR = 7.74, 95% CI: 6.48–9.25]. In the fully adjusted Model 4, the possibility of depression was 1,082% higher in Q3 [OR = 11.82, 95% CI: 9.81–14.25] and 953% higher in Q4 [OR = 10.53, 95% CI: 8.67–12.79]. Additionally, the RCS analysis confirmed the significant association between CMI and depression (Figure 2). Using a two-stage logistic regression model, we identified a CMI threshold of 0.594 (95% CI: 0.561–0.627). Below this threshold, the risk of depression increased sharply with rising CMI, while above the threshold, the correlation between CMI and depression risk plateaued (Supplementary Table 1). Individuals with a CMI of 0.594 or higher faced a 162% greater risk of depression in comparison to those with a CMI below 0.594 (OR = 2.62, 95% CI: 2.36–2.91).

**Table 2:** Association between CMI and depression (depressive symptoms and CESD-10 scores).

**CMI**	**Depression symptoms**	**CESD-10 score**
	**[OR (95% CI)]**	**[**β **(95% CI)]**
**Crude model (Model 1)**		
Continuous	1.08 (1.04, 1.11)	0.16 (0.07, 0.26)
**Categories**		
Quartile 1	1 (Ref)	0 (Ref)
Quartile 2	8.57 (7.17, 10.23)	3.81 (3.43, 4.2)
Quartile 3	10.04 (8.4, 11.99)	4.47 (4.09, 4.85)
Quartile 4	7.74 (6.48, 9.25)	3.59 (3.21, 3.97)
*P* for trend	< 0.001	< 0.001
**Adjusted model (Model 2)**		
Continuous	1.09 (1.05, 1.13)	0.19 (0.1, 0.29)
**Categories**		
Quartile 1	1 (Ref)	0 (Ref)
Quartile 2	8.64 (7.21, 10.35)	3.58 (3.21, 3.95)
Quartile 3	10.14 (8.46, 12.15)	4.21 (3.84, 4.59)
Quartile 4	8.04 (6.7, 9.64)	3.46 (3.09, 3.83)
*P* for trend	< 0.001	< 0.001
**Adjusted model (Model 3)**		
Continuous	1.09 (1.05, 1.13)	0.2 (0.1, 0.29)
**Categories**		
Quartile 1	1 (Ref)	0 (Ref)
Quartile 2	8.95 (7.46, 10.74)	3.6 (3.23, 3.96)
Quartile 3	10.7 (8.9, 12.85)	4.26 (3.89, 4.64)
Quartile 4	8.9 (7.38, 10.74)	3.61 (3.23, 3.99)
*P* for trend	< 0.001	< 0.001
**Adjusted model (Model 4)**		
Continuous	1.18 (1.12, 1.25)	0.34 (0.22, 0.46)
**Categories**		
Quartile 1	1 (Ref)	0 (Ref)
Quartile 2	9.38 (7.8, 11.27)	3.67 (3.3, 4.04)
Quartile 3	11.82 (9.8, 14.25)	4.45 (4.08, 4.83)
Quartile 4	10.53 (8.67, 12.79)	3.95 (3.56, 4.35)
*P* for trend	< 0.001	< 0.001

OR, odds ratio; CI, confidence interval.

Model 1: Unadjusted model.

Model 2: Adjusted for gender, age, marital status, Han ethnicity, education, and residence.

Model 3: Further adjusted for drink, smoke, social activities, diabetes, and hypertension.

Model 4: Further adjusted for TC, LDL, BUN, creatinine, CRP, uric acid, and cystatin C.

### Subgroup analyses

**Figure 3:**
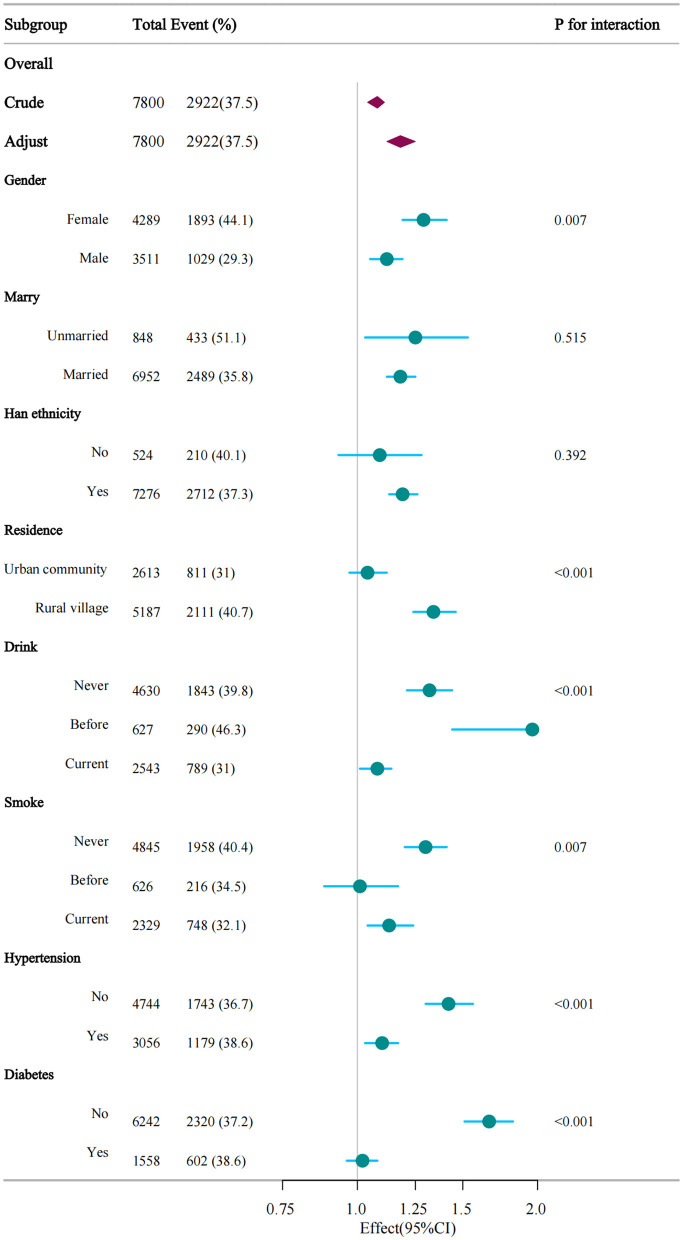
Verification of the association between CMI and depression by subgroup analyses. This figure illustrates the consistency of the association across different demographic and health subgroups, including gender, smoking status, alcohol consumption, diabetes, and hypertension. The analysis shows that the association between higher CMI and depressive symptoms remains significant across most subgroups, with variations in effect size by subgroup.

Interaction tests and subgroup analyses were performed to evaluate if the correlation between CMI and depression varied according to gender, marital status, ethnicity, residence location, alcohol use, smoking habits, hypertension, and diabetes (Figure 3 and Supplementary Table 5). The association between CMI and depression significantly differed by gender (*P* = 0.007), with each unit elevate in CMI increasing the risk of depression by 29% in women and 12% in men. Place of residence also significantly influenced the association between CMI and depression (*P* < 0.001), with each unit increase in CMI raising the likelihood of depression by 34% among rural participants and 4% in urban participants. The relationship between CMI and depressive symptoms was prominently affected by alcohol consumption status, with each unit rise in CMI linked to a 32% higher risk in non-drinkers, 96% in former drinkers, and 8% in current drinkers. Hypertension significantly modified the relationship between CMI and depression (*P* < 0.001). In non-hypertensive individuals, each unit increase in CMI elevated the risk of depression by 42%. Diabetes also significantly modified this association (*P* < 0.001), with each unit rise in CMI increasing the likelihood of depression by 66% in non-diabetic individuals (Supplementary Table 2).

### Sensitivity analyses

Table 3 reveals the results of the sensitivity analyses. After propensity score matching (5,592 participants, including 2,796 with depressive symptoms), each unit elevated in CMI was correlated with a 19% greater risk of depressive symptoms (95% CI: 1.11–1.27). Similarly, in the 2015–2016 CHARLS cohort (8,457 participants, 2,898 of whom had depressive symptoms), the relationship between CMI and depressive symptoms remained significant, with each unit rise in CMI correlated with a 17% higher risk (95% CI: 1.09–1.26). In addition, we conducted a sensitivity analysis by restricting the analysis to participants with complete data, thereby excluding the potential bias introduced by missing variables and ensuring the robustness of our results (OR = 1.34, 95% CI:1.24–1.44, *P* < 0.05).

**Table 3:** Sensitivity analysis of the association between CMI and depression.

**Analysis**	**Events/Total**	**OR (95% CI)**	***P*-value**
PSM^a^	2,796/5,592	1.19 (1.11, 1.27)	< 0.001
2015–2016 wave^b^	2,898/8,457	1.17 (1.09, 1.26)	< 0.001
2011–2012 wave^c^	2,226/5,820	1.34 (1.24–1.44)	< 0.001

PSM, propensity score matched; PA, pairwise algorithmic; OR, Odds Ratio; CI, Confidence Interval.

^a^Participants were matched for gender, age, ethnicity, education, marriage, residence, drink, smoke, social activities, hypertension, diabetes, TC, LDL, BUN, Creatinine, CRP, uric acid, and Cystatin C by propensity scores.

^b^Participants aged under 45 years were excluded, and participants lacking information on the CESD-10 questionnaire and lacking information on HEIGHT, WAIST, TG, and HDL-C were excluded, as well as participants lacking important covariates such as marital status, ethnicity information, education level, and place of residence information, resulting in the inclusion of 8,457 participants from the CHARLS cohort in the 2015–2016 wave participants, and multifactor regression analysis were performed according to model 4 in Table 2.

^c^Participants with complete data for all variables were included in the analysis (those with any missing variables were excluded). The multivariate analysis was adjusted for gender, age, ethnicity, education, marital status, place of residence, alcohol consumption, smoking status, social activities, hypertension, diabetes, total cholesterol (TC), low-density lipoprotein (LDL), blood urea nitrogen (BUN), creatinine, C-reactive protein (CRP), uric acid, and cystatin C.

## Discussion

In this cross-sectional study utilizing data from the 2011–2012 wave of the CHARLS cohort (*N* = 7,800), a robust association emerged between CMI and depressive symptoms in fully adjusted models. Notably, this association persisted after propensity score matching to address baseline confounding and was further validated in an independent CHARLS subsample, reinforcing result reliability. Subgroup stratification revealed differential vulnerability, with the highest odds ratios (ORs) for the CMI-depression link observed in females, rural residents, ever-alcohol consumers, never-smokers, and individuals without hypertension or diabetes. The amplified association in women may originate from sex-specific biological pathways. Estrogen's dual regulation of lipid metabolism and neuroinflammation (31) could create a synergistic pathway linking metabolic dysregulation to mood disorders. Mechanistically, postmenopausal estrogen decline correlates with visceral adiposity and microglial activation (32), thereby potentiating neuroinflammatory cascades that may drive depression pathogenesis in high-CMI individuals. Concurrently, gender-related psychosocial factors—including disproportionate caregiving responsibilities and culturally reinforced emotional suppression norms (26)—may compound physiological stress responses and subjective symptom reporting in women. Geographic disparities further modulated risk stratification. Rural residents exhibited heightened susceptibility, likely reflecting systemic barriers such as insufficient access to preventive metabolic screenings and mental health services (33). These structural gaps may delay early interventions, enabling subclinical metabolic disturbances to evolve into overt depression.

The substantial variations in sample sizes between subgroups may explain these differences, thus, propensity score matching was employed to equalize the baseline disparities.

As we all known, this is the foremost investigation of the association between CMI and depressive symptoms within the Chinese community of those in their middle to senior years. A cross-sectional study using NHANES 2011–2018 data reported findings similar to ours (9), demonstrating a prominent association between CMI and the possibility of depression [OR = 1.36, 95% CI: 1.16–1.59]. It is noteworthy that our study included participants aged 45 years or over, while the NHANES study incorporated those aged 20 years or over. However, its subgroup analysis revealed a stronger association between CMI and depression in participants aged 60 and above [OR = 1.48, 95% CI: 1.17–1.89].

A cross-sectional study using NHANES 2011–2014 data also yielded findings consistent with ours (8), showing a prominent association between CMI and the likelihood of depression [OR = 1.36, 95% CI: 1.16–1.59]. Subgroup analysis indicated a stronger association between CMI and depression in participants < 60 years of age [OR = 2.49, 95% CI: 1.92–3.23], although the association remained significant in participants over 60 years [OR = 2.21, 95% CI: 1.51–3.25], with no significant interaction between age groups (*P* = 0.666). The curvilinear relationship between CMI and depression perceived in this study aligns with our findings. However, we observed a saturation effect in the relationship between CMI and depression at CMI ≥ 0.594, where participants with CMI ≥ 0.594 had a 162% greater likelihood of depression in comparison to those with CMI < 0.594 [OR = 2.62, 95% CI: 2.36–2.91]. The saturation effect was not reported in this study, and the inflection points occurred at CMI = 0.9522 and CMI = 1.58, with ORs of 1.00, 1.87, and 2.05 for CMI ranges of < 0.9522, 0.9522–1.58, and ≥1.58, respectively. The CHARLS cohort specifically targeted middle-aged and older Chinese adults (aged ≥ 45 years), contrasting with the NHANES population that included participants as young as 20 years. Age-related metabolic alterations—particularly elevated visceral adiposity and insulin resistance (34)—could reduce the CMI threshold required to identify depression risk in aging populations. Ethnic disparities in metabolic profiles likely contribute to these variations. East Asian individuals, for example, demonstrate greater visceral fat accumulation at comparable BMI levels relative to Western populations (35, 36), a physiological distinction that might heighten the clinical relevance of lower CMI values in this demographic. Divergent comorbidity patterns and lifestyle behaviors further distinguish the cohorts. Notably, rural residents and female participants in our study displayed heightened CMI-depression correlations, suggesting subgroup-specific metabolic susceptibilities not observed in the NHANES cohort. Methodological distinctions also warrant consideration. The CHARLS assessment relied on the CESD-10 scale, whereas NHANES adopted the PHQ-9. This difference holds implications for threshold determination, as the CESD-10's emphasis on somatic manifestations (e.g., fatigue, appetite changes) overlaps with metabolic dysregulation phenotypes (37), potentially conflating biological and psychological endpoints. Furthermore, analytical discrepancies arose from covariate selection. Our models accounted for residence type (rural/urban) and social engagement—variables absent in NHANES datasets—introducing heterogeneity in spline-derived inflection points between studies.

A study involving nine cohorts from the Netherlands demonstrated an association between depression and metabolic changes (11). The study used proton nuclear magnetic resonance metabolomics to compare metabolic markers between depressed and non-depressed individuals, concluding that inferior HDL-C levels and superior triglyceride levels were prominently correlated with an increasing likelihood of depression (*p* < 0.05). The CMI, a recently introduced measure, is figured out by multiplying the WHtR by the TG/HDL-C (8, 9, 12), reflects both obesity and lipid levels. Studies have shown to be useful in identifying diabetes (7), erectile dysfunction (15), and predicting the progression of atherosclerosis (38).

There is a complex interplay between obesity, lipid metabolism disorders, and depression (39, 40). CMI, as an integrated measure of metabolic health, captures both visceral adiposity and lipid abnormalities, both of which are strongly associated with an increased risk of depression. A meta-analysis and systematic review found that individuals with abdominal obesity had a prominently greater possibility of depression in comparison to non-obese participants, with a relative risk (RR) of 1.38 (41). Additionally, a prospective cohort study in a male population reported that both overall obesity, evaluated by BMI, and abdominal obesity, measured by Visceral Adipose Tissue (VAT) area, were prominently associated with a greater occurrence of depressive symptoms. However, after multivariate modification for BMI and VAT, only VAT remained statistically significant in predicting the likelihood of developing depression in men. Lipid dysregulation also plays a gender-specific role in depression. In men, elevated LDL cholesterol levels are linked to an increased likelihood of depression, while in women, reduced HDL cholesterol levels are associated with a higher prevalence of depression (42, 43). Genetic factors may contribute to this relationship, as interactions between LDL cholesterol levels and serotonin transporter gene polymorphisms have been observed in men, further increasing their risk of depression (44). These findings underscore the critical role of lipid metabolism in the mental health of the older adults. A longitudinal study revealed that severe depression or anxiety symptoms predicted lower HDL cholesterol levels and increased abdominal obesity (45), both of which were not associated with symptom improvement. Among older patients, higher levels of LDL, total cholesterol, and triglycerides were prominently observed in those with depressive symptoms compared to their non-depressed counterparts (46). The prevailing view is that individuals suffering from depression experience elevated glucocorticoid levels due to chronic dysregulation of the hypothalamic-pituitary-adrenal (HPA) axis, which plays a pivotal role in stress response and metabolic regulation (47–49). Prolonged exposure to elevated glucocorticoids not only disrupts insulin's glycemic control but also induces insulin resistance, leading to persistent hyperglycemia and excessive fat accumulation. This metabolic dysregulation further contributes to chronic inflammation, promoting the release of pro-inflammatory cytokines such as IL-6 and TNF-α, which have been implicated in both depression onset and severity (50). Furthermore, cortisol-induced metabolic disturbances can impair neuroplasticity by affecting hippocampal structure and function, thereby exacerbating depressive symptoms (51). Studies suggest that persistent glucocorticoid elevation is associated with reduced hippocampal volume (52), synaptic dysfunction, and neurotransmitter imbalances, particularly involving serotonin and dopamine (53). These changes not only reinforce depressive symptoms but also create a bidirectional relationship between metabolic dysregulation and mood disorders, where depression itself perpetuates metabolic abnormalities (50). This pathophysiological interplay highlights the potential of metabolic-targeted interventions in disrupting the self-perpetuating cycle between elevated CMI and depression progression. As metabolic dysregulation, particularly lipid abnormalities, plays a critical role in depression risk, nutritional strategies, such as the Mediterranean diet and other anti-inflammatory diets, have demonstrated robust evidence in mitigating depression risk (54). A prospective cohort study reported that higher adherence to the Dutch Healthy Diet correlated with a notably reduced incidence of depressive symptoms (55). In rural Chinese populations, diets high in vegetables, fruits, and lean animal proteins were associated with a lower risk of late-life depression, whereas pro-inflammatory dietary patterns exacerbated susceptibility (56). Comprehensive meta-analyses consistently indicate that structured exercise programs substantially ameliorate depressive symptoms across various demographics (57). The antidepressant properties of exercise are mediated via multifaceted pathways, including neurotransmitter modulation (serotonin, dopamine, and GABA) and BDNF upregulation, fostering neuroplasticity and stress resilience (58, 59). Both aerobic training and mind-body practices, performed 3–5 times weekly at moderate intensity over 4–16 weeks, demonstrate consistent efficacy in alleviating depressive symptoms (60). Metformin, a first-line antidiabetic agent, has exhibited neuroprotective and antidepressant potential by attenuating depression-like phenotypes and metabolic impairments in preclinical models (61). A nationwide cohort study revealed that long-term metformin therapy was associated with a decreased risk of incident depression (62). The antidepressant-like actions of metformin are hypothesized to involve AMP-activated protein kinase (AMPK) activation in the brain, a central regulator of energy metabolism and neuroinflammation (63). These mechanisms highlight the importance of addressing both metabolic and psychological aspects of health in older adults. Monitoring metabolic health, including CMI, should be a routine part of depression screening in clinical settings. Early identification of individuals with elevated CMI could lead to more effective intervention strategies targeting both metabolic dysregulation and depressive symptoms.

## Limitations

A major strength of our study is that it represents the first large-scale cross-sectional analysis based on the CHARLS cohort. However, several limitations must be acknowledged. Due to the cross-sectional design, causal relationships between CMI and depression cannot be established, highlighting the need for well-conducted prospective cohort studies to address this limitation. While our multivariate models adjusted for known confounders such as smoking status, alcohol use, and urban/rural residence, residual confounding may persist due to unmeasured factors, including dietary patterns, physical activity, sleep disorders, and chronic exposure to environmental pollutants. Furthermore, given that the CHARLS database primarily targets individuals aged 45 and older, the findings of this study should be interpreted and generalized with caution.

Additionally, as this study relied on publicly available data from the CHARLS database, there are limitations in the medication data. Participants were only asked about antidepressant use if they self-reported having “emotional or psychiatric problems” or if a doctor had previously informed them of such issues. Consequently, only a small proportion of participants (49) reported using antidepressants, and only one participant in the final sample of 7,800 was recorded as using antidepressants. This may not fully reflect the true extent of antidepressant use in individuals with depressive symptoms. Moreover, since the CHARLS database does not provide detailed medication data, we were unable to account for other medications, such as fenofibrate or omega-3, that may influence the relationship between CMI and depression. The absence of detailed medication data limits our ability to explore the potential effects of pharmacological treatments on the study outcomes. Future research with more detailed medication data could help clarify these potential effects.

## Conclusion

In summary, our research suggests that higher CMI levels are strongly linked to elevated CESD-10 scores and a greater possibility of depression among Chinese people of middle age and beyond. This relationship is particularly evident among women, rural dwellers, former drinkers, non-smokers, and those without hypertension or diabetes. Nevertheless, further extensive prospective research is required to validate these findings.

## Data Availability

The raw data supporting the conclusions of this article will be made available by the authors, without undue reservation.
