# Supplementary material for: Curvilinear association between cardiometabolic index and depressive symptoms in individuals aged 45 and older: a cross-sectional study of CHARLS
Source: Front Public Health. 2025 Mar 19;13:1534302. doi: 10.3389/fpubh.2025.1534302 (PMC11963158; doi:10.3389/fpubh.2025.1534302)
Supplement: Supplementary file 1 [file Table_1.docx]

Supplementary Table 1

|  | **OR (95%CI)** | **P value** |
| --- | --- | --- |
| **Turning point** | 0.594 (0.561,0.627) |  |
| **CMI＜0.594** | 1(Ref) |  |
| **CMI≥0.594** | 2.62 (2.36~2.91) | <0.001 |
| Likelihood Ratio test | | <0.001 |
| Non-linearity tested by quadratic term | | <0.001 |
| Non-linearity tested by cubic spline term (LRT) | | <0.001 |

OR:Odds Ratio; CI:Confidence Interval.

Inflection point analysis of the restricted cubic spline fit between CMI and depression (2-stage logistic multiple regression analysis adjusting for sex, age, ethnicity, education, marriage, place of residence, alcohol consumption, smoking, social activity, hypertension, diabetes, TC, LDL, blood pressure, creatinine, CRP, uric acid and cystatin C).
