# Supplementary material for: Curvilinear association between cardiometabolic index and depressive symptoms in individuals aged 45 and older: a cross-sectional study of CHARLS
Source: Front Public Health. 2025 Mar 19;13:1534302. doi: 10.3389/fpubh.2025.1534302 (PMC11963158; doi:10.3389/fpubh.2025.1534302)
Supplement: Supplementary file 3 [file Table_3.docx]

**Supplementary Table 3**

Baseline Characteristics of Participants After Propensity Score Matching by Depression Status.

| **Variables** | **Total (n = 5,592)** | **Non-depressed (n = 2,796)** | **Depressed (n = 2,796)** | **p** |
| --- | --- | --- | --- | --- |
| **Gender** |  |  |  | 0.505 |
| Female | 3,558 (63.6) | 1,791 (64.1) | 1,767 (63.2) |  |
| Male | 2,034 (36.4) | 1,005 (35.9) | 1,029 (36.8) |  |
| **Age** | 59.2 ± 8.7 | 59.2 ± 8.9 | 59.2 ± 8.6 | 0.825 |
| **Marriage** |  |  |  | 0.628 |
| Unmarried | 700 (12.5) | 356 (12.7) | 344 (12.3) |  |
| Married | 4,892 (87.5) | 2,440 (87.3) | 2,452 (87.7) |  |
| **Han ethnicity** |  |  |  | 0.789 |
| No | 375 ( 6.7) | 185 (6.6) | 190 (6.8) |  |
| Yes | 5,217 (93.3) | 2,611 (93.4) | 2,606 (93.2) |  |
| **Education** |  |  |  | 0.909 |
| Below primary school level | 3,129 (56.0) | 1,571 (56.2) | 1,558 (55.7) |  |
| Primary school | 1,216 (21.7) | 600 (21.5) | 616 (22) |  |
| Junior high school | 908 (16.2) | 451 (16.1) | 457 (16.3) |  |
| High school and above | 339 ( 6.1) | 174 (6.2) | 165 (5.9) |  |
| **Residence** |  |  |  | 0.882 |
| Urban community | 1,605 (28.7) | 805 (28.8) | 800 (28.6) |  |
| Rural village | 3,987 (71.3) | 1,991 (71.2) | 1,996 (71.4) |  |
| **Social activities** |  |  |  | 0.728 |
| Passive | 2,967 (53.1) | 1,490 (53.3) | 1,477 (52.8) |  |
| Positive | 2,625 (46.9) | 1,306 (46.7) | 1,319 (47.2) |  |
| **Hypertension** |  |  |  | 0.566 |
| No | 3,387 (60.6) | 1,704 (60.9) | 1,683 (60.2) |  |
| Yes | 2,205 (39.4) | 1,092 (39.1) | 1,113 (39.8) |  |
| **Diabetes** |  |  |  | 0.485 |
| No | 4,457 (79.7) | 2,239 (80.1) | 2,218 (79.3) |  |
| Yes | 1,135 (20.3) | 557 (19.9) | 578 (20.7) |  |
| **Alcohol consumption** |  |  |  | 0.341 |
| Never | 3,500 (62.6) | 1,774 (63.4) | 1,726 (61.7) |  |
| Before | 541 ( 9.7) | 258 (9.2) | 283 (10.1) |  |
| Current | 1,551 (27.7) | 764 (27.3) | 787 (28.1) |  |
| **Smoking status** |  |  |  | 0.529 |
| Never | 3,703 (66.2) | 1,871 (66.9) | 1,832 (65.5) |  |
| Before | 419 ( 7.5) | 203 (7.3) | 216 (7.7) |  |
| Current | 1,470 (26.3) | 722 (25.8) | 748 (26.8) |  |
| **TC(mg/dl)** | 194.8 ± 39.1 | 194.5 ± 39.3 | 195.0 ± 39.0 | 0.629 |
| **LDL(mg/dl)** | 117.2 ± 35.1 | 117.0 ± 35.1 | 117.4 ± 35.1 | 0.671 |
| **Weight(kg)** | 57.8 ± 11.3 | 58.5 ± 11.3 | 57.1 ± 11.3 | < 0.001 |
| **BUN(mg/dl)** | 15.6 ± 4.4 | 15.6 ± 4.4 | 15.6 ± 4.4 | 0.453 |
| **Creatinine(mg/dl)** | 0.8 ± 0.2 | 0.8 ± 0.2 | 0.8 ± 0.2 | 0.616 |
| **Uric Acid(mg/dl)** | 4.3 ± 1.2 | 4.3 ± 1.2 | 4.3 ± 1.2 | 0.89 |
| **Cystatin C(mg/dl)** | 1.0 ± 0.2 | 1.0 ± 0.2 | 1.0 ± 0.2 | 0.937 |

Abbreviations: OR, odds ratio; CI, confidence interval; TC, total cholesterol; LDL, low-density lipoprotein; BUN, blood urea nitrogen.
