# Supplementary material for: Curvilinear association between cardiometabolic index and depressive symptoms in individuals aged 45 and older: a cross-sectional study of CHARLS
Source: Front Public Health. 2025 Mar 19;13:1534302. doi: 10.3389/fpubh.2025.1534302 (PMC11963158; doi:10.3389/fpubh.2025.1534302)
Supplement: Supplementary file 4 [file Table_4.docx]

**Supplementary Table 4**

Missing Data in Variables

| Variable | Miss.frequency | Miss.percentage% |
| --- | --- | --- |
| Age,y | 0 | 0 |
| BUN(mg/dl) | 1 | 0.0128 |
| Creatinine(mg/dl) | 10 | 0.1282 |
| CRP(mg/dl) | 1 | 0.0128 |
| Cystatin C(mg/dl) | 1964 | 25.1795 |
| Uric Acid(mg/dl) | 1 | 0.0128 |
| CESD-10 | 0 | 0 |
| CMI | 0 | 0 |
| Depression | 0 | 0 |
| Diabetes | 0 | 0 |
| Alcohol consumption | 0 | 0 |
| Education | 0 | 0 |
| Gender | 0 | 0 |
| HDL(mmol/L) | 0 | 0 |
| Hypertension | 0 | 0 |
| LDL(mg/dl) | 16 | 0.2051 |
| Marriage | 0 | 0 |
| Han ethnicity | 0 | 0 |
| Residence | 0 | 0 |
| Smoking status | 0 | 0 |
| Social activities | 0 | 0 |
| TC(mg/dl) | 2 | 0.0256 |
| TG(mmol/L) | 0 | 0 |
| WHtR | 0 | 0 |
