# Supplementary material for: Curvilinear association between cardiometabolic index and depressive symptoms in individuals aged 45 and older: a cross-sectional study of CHARLS
Source: Front Public Health. 2025 Mar 19;13:1534302. doi: 10.3389/fpubh.2025.1534302 (PMC11963158; doi:10.3389/fpubh.2025.1534302)
Supplement: Supplementary file 5 [file Table_5.docx]

**Supplementary Table 5**

**Subgroup analyses**

| Subgroup | n.total | n.event_% | OR,95CI% | P-value | P.for.interaction |
| --- | --- | --- | --- | --- | --- |
| **Gender** |  |  |  |  |  |
| Female | 4,289 | 1,893 (44.1) | 1.29 (1.19~1.41) | <0.001 | 0.007 |
| Male | 3,511 | 1,029 (29.3) | 1.12 (1.05~1.19) | <0.001 |  |
| **M**arriage |  |  |  |  |  |
| Unmarried | 848 | 433 (51.1) | 1.25 (1.03~1.53) | 0.024 | 0.515 |
| Married | 6,952 | 2,489 (35.8) | 1.18 (1.12~1.25) | <0.001 |  |
| **Han ethnicity** |  |  |  |  |  |
| No | 524 | 210 (40.1) | 1.09 (0.93~1.28) | 0.299 | 0.392 |
| Yes | 7,276 | 2,712 (37.3) | 1.19 (1.13~1.26) | <0.001 |  |
| **Residence** |  |  |  |  |  |
| Urban community | 2,613 | 811 (31) | 1.04 (0.97~1.12) | 0.241 | <0.001 |
| Rural village | 5,187 | 2,111 (40.7) | 1.34 (1.24~1.46) | <0.001 |  |
| **A**lcohol consumption |  |  |  |  |  |
| Never | 4,630 | 1,843 (39.8) | 1.32 (1.21~1.44) | <0.001 | <0.001 |
| Before | 627 | 290 (46.3) | 1.96 (1.44~2.67) | <0.001 |  |
| Current | 2,543 | 789 (31) | 1.08 (1.01~1.14) | 0.02 |  |
| **S**moking status |  |  |  |  |  |
| Never | 4,845 | 1,958 (40.4) | 1.3 (1.2~1.41) | <0.001 | 0.007 |
| Before | 626 | 216 (34.5) | 1.01 (0.88~1.17) | 0.864 |  |
| Current | 2,329 | 748 (32.1) | 1.13 (1.04~1.24) | 0.005 |  |
| **Hypertension** |  |  |  |  |  |
| No | 4,744 | 1,743 (36.7) | 1.42 (1.3~1.56) | <0.001 | <0.001 |
| Yes | 3,056 | 1,179 (38.6) | 1.1 (1.03~1.17) | 0.004 |  |
| **Diabetes** |  |  |  |  |  |
| No | 6,242 | 2,320 (37.2) | 1.66 (1.51~1.82) | <0.001 | <0.001 |
| Yes | 1,558 | 602 (38.6) | 1.02 (0.96~1.08) | 0.541 |  |
